# Distraction by a cognitive task has a higher impact on electrophysiological measures compared with conditioned pain modulation

**DOI:** 10.1186/s12868-020-00604-1

**Published:** 2020-12-07

**Authors:** A. T. L. Do, E. K. Enax-Krumova, Ö. Özgül, L. B. Eitner, S. Heba, M. Tegenthoff, C. Maier, O. Höffken

**Affiliations:** 1 Department of Neurology, Ruhr-University Bochum, BG University Hospital Bergmannsheil gGmbH, Bürkle-de-la-Camp-Platz 1, 44789 Bochum, Germany; 2 Department of Pain Medicine, Ruhr-University Bochum, BG University Hospital Bergmannsheil gGmbH, Bürkle-de-la-Camp-Platz 1, 44789 Bochum, Germany; 3 Department of Neuropaediatrics, University Children’s Hospital, Ruhr-University Bochum, Alexandrinenstraße 5, 44791 Bochum, Germany

**Keywords:** Painful cutaneous electrical stimulation, Conditioned pain modulation, Cognitive distraction, Pain mechanisms

## Abstract

**Background:**

Conditioned pain modulation (CPM) evaluates the effect of a painful conditioning stimulus (CS) on a painful test stimulus (TS). Using painful cutaneous electrical stimulation (PCES) as TS and painful cold water as CS, the pain relief was paralleled by a decrease in evoked potentials (PCES-EPs). We now aimed to compare the effect of CPM with cognitive distraction on PCES-induced pain and PCES-EP amplitudes.

**Methods:**

PCES was performed using surface electrodes inducing a painful sensation of 60 (NRS 0–100) on one hand. In a crossover design healthy subjects (included: n = 38, analyzed: n = 23) immersed the contralateral hand into 10 °C cold water (CS) for CPM evaluation and performed the 1-back task for cognitive distraction. Before and during the CS and 1-back task, respectively, subjects rated the pain intensity of PCES and simultaneously cortical evoked potentials were recorded.

**Results:**

Both CPM and cognitive distraction significantly reduced PCES-EP amplitudes (CPM: 27.6 ± 12.0 μV to 20.2 ± 9.5 μV, cognitive distraction: 30.3 ± 14.2 µV to 13.6 ± 5.2 μV, p < 0.001) and PCES-induced pain (on a 0–100 numerical rating scale: CPM: 58 ± 4 to 41.1 ± 12.3, cognitive distraction: 58.3 ± 4.4 to 38.0 ± 13.0, p < 0.001), though the changes in pain intensity and PCES-amplitude did not correlate. The changes of the PCES-EP amplitudes during cognitive distraction were more pronounced than during CPM (p = 0.001).

**Conclusions:**

CPM and cognitive distraction reduced the PCES-induced pain to a similar extent. The more pronounced decrease of PCES-EP amplitudes after distraction by a cognitive task implies that both conditions might not represent the general pain modulatory capacity of individuals, but may underlie different neuronal mechanisms with the final common pathway of perceived pain reduction.

## Background

The processing of nociceptive information is modulated by different endogenous mechanisms, which can be antinociceptive or pronociceptive [1, 2]. The conditioned pain modulation (CPM) evaluates the effect of a noxious conditioning stimulus (CS) on a noxious test stimulus (TS), as a surrogate for the function of the descending pain inhibitory pathways [1, 2]. A pronounced CPM-effect was shown to be inversely related to pain frequency among healthy individuals [3], whereas a reduced CPM-effect has been found in different chronic pain states [4, 5] and seems to represent a risk for chronic postoperative pain [6–8].

Several functional magnetic resonance imaging (fMRI) studies examined the neural mechanism of CPM. Here, cortical and subcortical brain regions are considered to control the descending modulatory pathways [9–12]. Dependent on which stimuli were used for TS and CS, increased MR signal intensities during each TS decreased in presence of CS in brain regions such as the caudal subdivision of the spinal trigeminal nucleus, the region of the subnucleus reticularis dorsalis and the dorsolateral pons near the parabrachial nucleus, the anterior cingulate, orbitofrontal and lateral prefrontal cortices, the amygdala, the primary and secondary somatosensory cortices, supplementary motor area and posterior insula [13–15].

Although CPM has been extensively studied both in animals [16, 17]⁠ and humans using psychophysiological and electrophysiological methods [18–24], it is still under debate whether the pain reduction during CS results solely from the descending noxious inhibitory pathways. Pain modulation can be achieved also by cognitive factors like attention and distraction [25–27]. Postsurgical pain is reported as more intense when patients are attending to it [28]; in contrast, listening to music [29–31], therapeutic play interventions [32] and animal-assisted treatments [33] for children can reduce postoperative pain intensity, presumably by distraction. Generally, subjects’ attention has to be actively directed elsewhere to avoid that painful stimuli prevail over competing non-painful ones [34–37].⁠ Interestingly, both CS and distraction by a cognitive visual task reduced pain intensity of heat stimuli in an additive manner [38].

Data including also objective parameters as readout to compare both pain modulating paradigms are missing. Therefore, we used a CPM paradigm, which we have recently introduced, based on both pain intensity and amplitudes of evoked cortical potentials after painful cutaneous electrical stimulation (PCES-EPs) in healthy subjects by hand immersion in painful cold water as CS [39]. Having an additional objective electrophysiological parameter as readout would make it possible to detect potential differences in signal processing and would also validate the utility of this potential objective biomarker. Hence, we aimed to compare the CPM effect using this paradigm with the pain relief during distraction by a cognitive 1-back task, which is commonly used to assess working memory capacity [34] and was shown to influence pain perception [40–42]. Hereby, we analyzed differences in both subjective pain ratings and objective electrophysiological readouts. Additionally, we examined whether pain intensity of the CS as well as perceived severity grade of the cognitive task and the anger felt about doing a mistake correlated with the changes during both interventions, respectively.

## Methods

### Study design and subjects

After approval by the Ethics Committee of the Medical Faculty of the Ruhr University Bochum, Germany (registration nr. 16-5733) 38 healthy subjects (22 females, 16 males, age 22.2 ± 2.4 years) were recruited after informed consent. All experiments were performed in accordance with relevant guidelines and regulations. The study was conducted in the Department of Neurology, University Hospital Bergmannsheil Bochum, Germany, between October 2016 and April 2017.

**Fig. 1:**
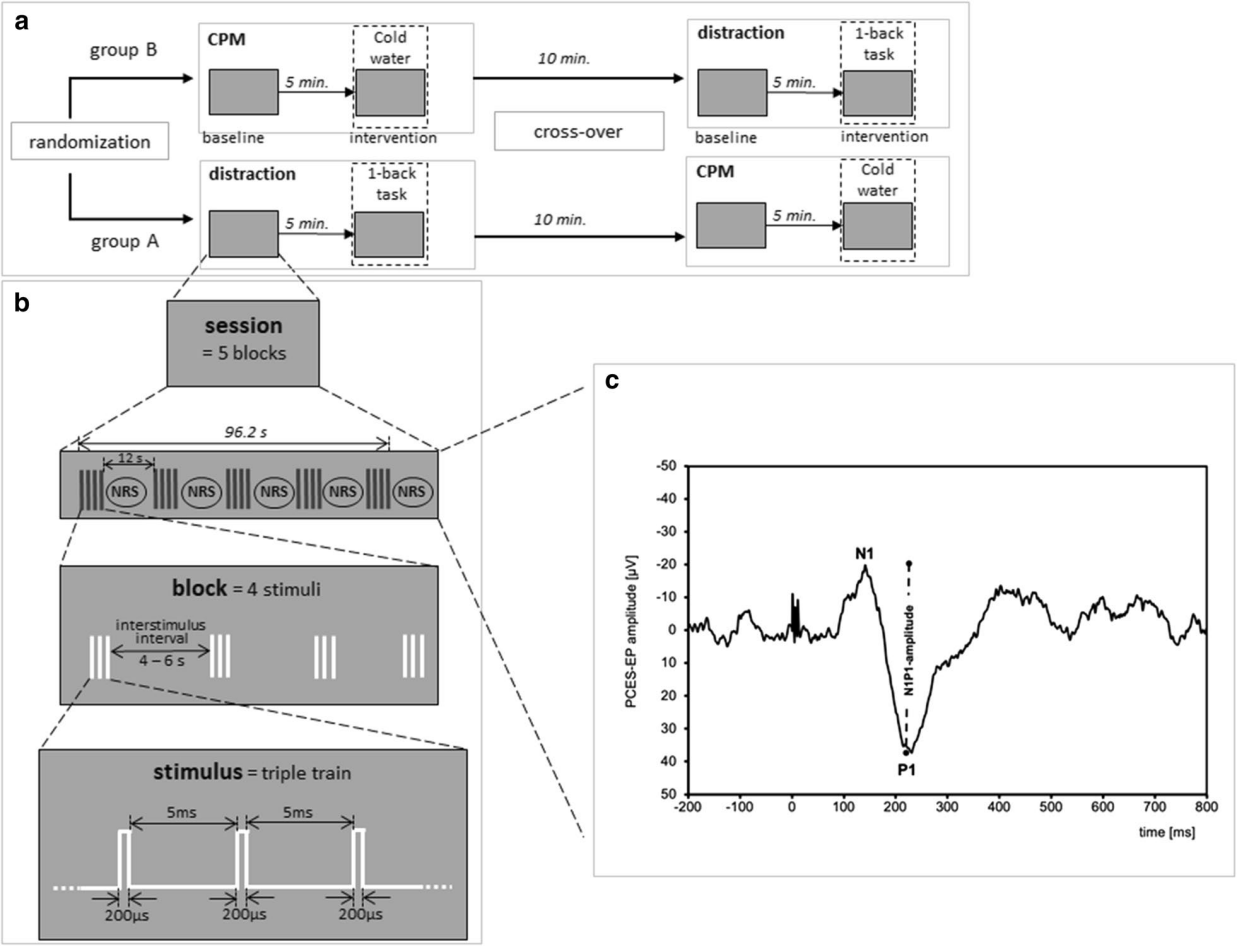
Study design. **a** Timeline of experimental procedure, **b** paradigm for electrical stimulation and **c** evoked potential after painful cutaneous electrical stimulation (PCES-EP) with N1 and P1 peaks recorded over Cz of one subject

The study was designed as a randomized cross-over study and the subjects were randomized to two groups (Fig. 1a). In group A at first we assessed the CPM and proceeded with the distraction by cognitive task afterwards. For group B, the first intervention was the distraction by cognitive task, and then the assessment of CPM was performed.

Exclusion criteria were insufficient German language skills, pain disorders, nerve injuries, neuropathy and any other neurological disease as well as intake of pain modulating drugs (in the last 2 weeks) or transdermal application of medication (in the last 6 weeks or more than seven days in the last 4 months). Further, we excluded subjects with diagnosis of epilepsy, psychiatric disease and/or circulatory disorders.

During the experiment, the subjects sat in an upright comfortable arm-chair in an air-conditioned room and were instructed to relax and avoid movements, especially of the head and the upper limbs, to prevent artifacts during EEG recording. However, we had to exclude four subjects due to insufficient recording signal caused by electrode malfunction and further eleven subjects because of the absence of enough detectable potentials during the intervention cold water-immersion or performing n-back task caused by severe movement artifacts or increased noise in signal processing. After all, we included 23 healthy subjects (10 females, 13 males, age 22.3 ± 2.7 years), randomized in two groups: the first included 12 subjects (6 females, 6 males, age 21.9 ± 2.8 years) and the second one 11 subjects (4 females, 7 males, age 22.8 ± 2.3 years).

### Painful cutaneous electrical stimulation (PCES)

For the cutaneous electrical stimulation three planar custom-built concentric electrodes were fixed in a triangular formation on the radial dorsum of the right hand in a distance of 1.5 to 2 cm to each other. The electrodes were connected in parallel to a stimulator (Digitimer DS7A), thus, the given current intensity was proportionally distributed over all electrodes (for a detailed description see [39]⁠). Each electrical stimulus, applied simultaneously by all three stimulation electrodes, consisted of a train of three monopolar square waves (200 μs duration, 5 ms inter-wave interval within the triple train). We applied 20 stimuli in 5 blocks with a variable inter-train interval of 4–5 s in a pseudo-randomized manner, with a fixed inter-block interval of 12 s (see Fig. 1b). We chose the method of a stable positioning in contrast to shifting between the stimulation parts as this would have led to multiple determination of the necessary stimulus intensity.

First, prior to both interventions we determined the individual detection thresholds (DT) for cutaneous electrical stimuli as well as the corresponding pain thresholds (PT) by increasing current intensities starting with 0.2 mA steps until the subjects reported a (mostly lightly tingling) sensation (DT) or a pinprick-like pain (PT), respectively. Then we proceeded in 0.1 mA steps in a randomized order above or below the supposed thresholds until the subjects reported a stable perception.

The stimulation intensity was adjusted to subjects’ individual pain intensity corresponding to 60 on the 101-point numerical rating scale (NRS, 0 = no pain, 100 = maximum pain imaginable) by increasing the current intensities starting with 0.5 mA steps and continuing, after subjects rated the pain as 50, with 0.1 mA steps.

The effect of the intervention (CPM or distraction, see below) was assessed with stimulation intensity, determined as described above, applying 20 stimuli before and 20 during the intervention (CS and 1-back-task, respectively, see below).

#### Pain ratings as a subjective readout

The pain perceived during PCES was reported by the participants after every 4 stimuli (= 1 block) as a rating on the 101-point numerical rating scale (NRS, 0 = no pain, 100 = maximum pain imaginable), resulting in a total of 5 ratings per session (compare Fig. 1). The *PCES-induced pain* was calculated as mean of the 5 ratings during PCES*.*

#### Cortical potentials as an electrophysiological readout

Cortical potentials evoked after painful cutaneous electrical stimulation (PCES-EPs) were recorded above Cz according to the international 10–20 system with reference to linked earlobes (A1–A2), as previously described [39] and stored for offline analysis (Brain Amp, Brain Products, Germany; Bandwith: 1 Hz–1 kHz; digitization sampling rate: 5 kHz) (see Fig. 1c). Impedances were kept below 5 kΩ. PCES-EPs were analyzed in sweeps from 200 ms before and 800 ms after every stimulus onset using Vision Recorder Version 1.03 as previously described in detail [39]. In accordance with previous studies [37, 43, 44], the first sweep was rejected to avoid bias by initial startling response. After averaging PCES-EPs, we analyzed amplitudes of N1-to-P1-peak of PCES-EPs (see Fig. 1c). Subjects with recordings with a high artefact overlap due to muscle excitation or loosening of the electrodes were manually identified and excluded.

### Intervention

#### Conditioned pain modulation (CPM)

For assessment of the CPM we used PCES on the right hand as TS and immersion of the left hand in cold water as CS. Therefore, subjects immersed their left hand into cold water bath with a temperature of 10 °C, which was controlled immediately before immersion of the hand using a digital thermometer. After 20 s of CS application, the first PCES started. Subjects dragged out their hand immediately after the last stimulus. Subjects rated the pain intensity induced by the PCES repeatedly on the 101-point NRS after each block (see “Pain ratings as a subjective readout”). Additionally, subjects rated the pain induced by cold water itself, on the 101-point NRS as well.

We calculated the CPM-effect based on the changes of the pain intensity and the PCES-amplitude as follows:

- The CPM-effect_PAIN_ was defined as difference between the mean of five pain ratings during CS and the mean of five pain ratings before CS (baseline).
- CPM-effect_AMPLITUDE_ was calculated as ratio between the amplitude of the averaged PCES-EP during CS and the amplitude of the averaged PCES-EP at baseline.

A difference < 0 for CPM-effect_PAIN_ represents an efficient pain inhibition [21, 45]. Analogously, a ratio < 1 for CPM-effect_AMPLITUDE_ indicated an efficient pain inhibition.

#### Distraction by a cognitive task

For the distraction by a cognitive task we used the well-established 1-back version of the n-back task that requires continuous updating of representations in working memory and response selection [40]. Before starting the 1-back task, subjects were introduced to the test setting. Additionally, instructions were displayed on the screen during the 1-back task. During this task, participants sat in front of a monitor (16-inch diagonal, resolution 1024 × 768 pixel) on which a stream of 85 lower-case letters (c, h, k, m, p, s, t, w, y) was presented with a frequency of 1 Hz. The task included 10 target stimuli and 75 nontarget stimuli presented in a random order. The letters appeared one at a time and in white Arial font on a black background in the center of the screen. The size of the letters was 21% of the monitor height. Each letter was presented for 500 ms, followed by a 500 ms blank screen. The distance between the subjects’ eyes and the monitor was about 95 cm. During presentation of the blank screen, participants were instructed to report whether the letter currently on screen matched the letter presented one letter ago; they indicated their response using two separate foot switches (left foot on foot switch “1” for “the current letter was presented 1 letter ago”, right one on foot switch “2” for “the current letter was not presented 1 letter ago”). Subjects were instructed to use their feet gently enough to avoid movement artifacts, yet strong enough to activate the foot switch. The first letter was displayed on the screen ten seconds before application of the first PCES, and the last letter appeared simultaneously with the last PCES. After every block the stream of letters stopped for 9 to 12 s so that the participants had time to report the current PCES-induced pain.

Analogous to the CPM assessment, the changes in PCES-induced pain intensity and PCES-evoked cortical potential were calculated each as difference and ratio between the values prior and during the intervention.

After completing the 1-back task, the subjects reported the perceived severity grade of the task and the anger felt while doing a mistake during the task as a rating on an 11-point numerical rating scale (0 = very easy task/no anger, 10 = very difficult task/very angry).

### Statistical analysis

In order to exclude effects of the randomization sequence (group A and B), in terms of carry-over effect, we have performed a preliminary test, calculating the sum of the values measured in the two interventions for each subject and comparing across the two sequence groups by means of unpaired t-test, according to the experts’ recommendations [46]. After exclusion of carry-over effects, we performed repeated measures ANOVA with PCES-induced pain and PCES-EP amplitude as dependent variables, and within-subjects factors “time” (before and during intervention) and “intervention” (CPM and distraction).

The changes of PCES-induced pain (differences) and PCES-EP-amplitudes (ratios) during CPM and distraction were compared using two-tailed paired t-tests.

Using Pearson correlation analysis, we correlated differences for PCES-induced pain to the ratios for PCES-EP amplitudes during both CPM and cognitive distraction. Additionally, we also correlated the difference for PCES-induced pain and ratios for PCES-amplitudes to the pain intensity rating of the cold water test during CPM session, and to the perceived severity grade of the 1-back task during the distraction, respectively, as well as with the PSQ-score (separately for both CPM and cognitive distraction). Due to the high number of correlations computed (n = 12), we adjusted the alpha-level for the correlations analysis using a Bonferroni-correction for multiple comparisons, resulting in a p < 0.004 for a significant correlation.

## Results

### Participants and group effects

Both randomization groups A and B did not differ in their age or gender distribution. After excluding an influence of the group affiliation during randomization (sequence of CPM and distraction as intervention) on both pain intensity and amplitudes of PCES-evoked potentials data from both groups were pooled for all analyses.

### Detection threshold, pain threshold and stimulation intensity

The mean detection threshold for the electrical cutaneous stimulation for perceiving any sensation was at 0.8 ± 0.2 mA and the main pain threshold for perceiving a pinprick sensation for the first time was 1.0 ± 0.3 mA. The mean stimulation intensity to induce a pain intensity corresponding to 60 on the NRS (0–100) was 7.1 ± 5.3 mA in the first intervention and 8.0 ± 5.0 mA in the second intervention (p < 0.001).

### Changes in pain intensity and cortical potentials during intervention

Group means and standard deviation are reported in Table 1.

**Table 1:** PCES-induced pain ratings and amplitudes of the PCES-evoked potentials as well as effects of the intervention (for pain ratings difference between baseline and during intervention, values < 0 implicate reduction of the pain rating during intervention; for amplitudes ratios between baseline and during intervention, values < 1 implicate reduction of the pain rating during intervention)

	Conditioned pain modulation		Distraction by a cognitive task	
	PCES-EP amplitude [µV, MW ± SD (range)]	PCES pain [NRS 0–100, MW ± SD (range)]	PCES-EP amplitude [µV, MW ± SD (range)]	PCES pain [NRS 0–100, MW ± SD (range)]
At baseline	27.6 ± 12.0 (8.7 … 57.7)	58.1 ± 4.5 (50 … 72)	30.3 ± 14.2 (18.5 … 84.6)	58.3 ± 4.4 (48 … 66)
During intervention	20.2 ± 9.5 (9.4 …. 50.6)	41.1 ± 12.3 (14 … 60)	13.6 ± 5.2 (6.3 … 26.6)	38.0 ± 13.0 (14….70)
Effect	0.76 ± 0.23 (0.45….1.24)	− 17.1 ± 13.0 (− 47 …. 0)	0.49 ± 0.20 (0.22…0.84)	− 20.3 ± 11.7 (− 44 …. 6)

#### Effects of CPM

**Fig. 2:**
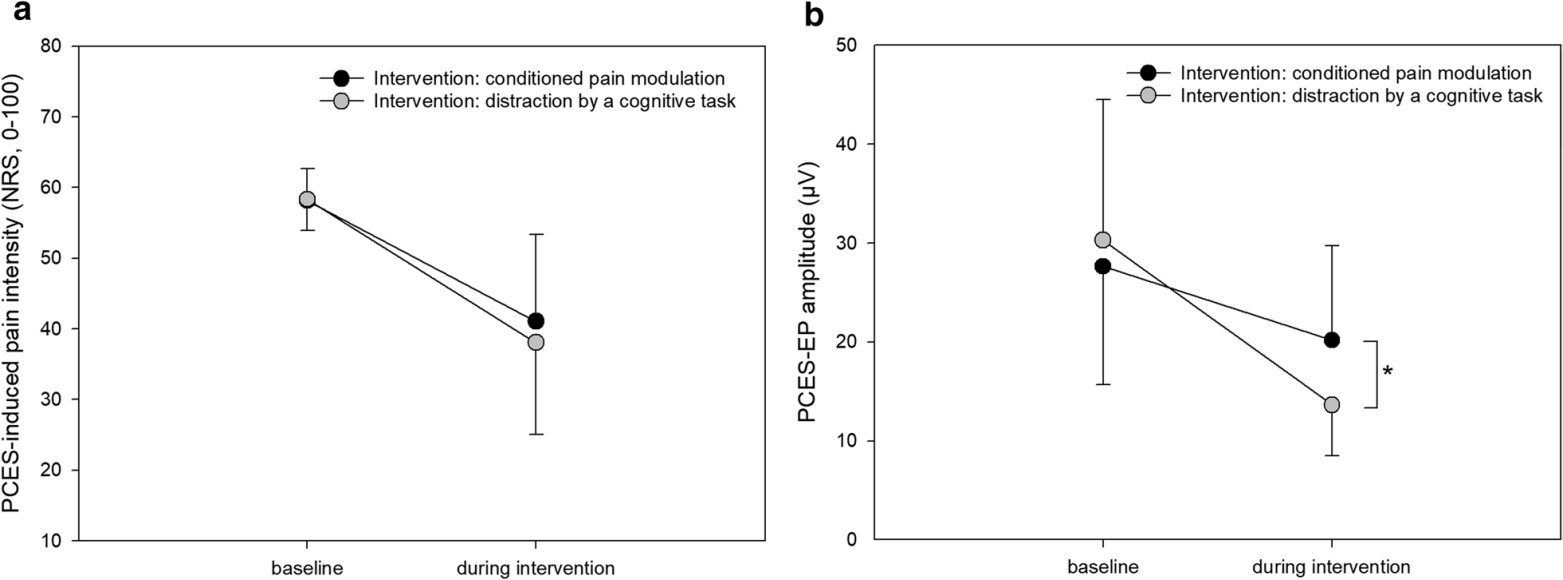
Effects of conditioning pain modulation and distraction by a cognitive task on pain intensity and evoked potential after painful cutaneous electrical stimulation (PCES) at baseline and during both interventions. **a** Changes in PCES-induced pain intensity and **b** amplitudes of the evoked potential after painful cutaneous electrical stimulation (PCES-EP). Data are presented as mean ± standard deviation. * p = 0.001 for ANOVA with within factors “time” (“at baseline” and “during intervention”) and “intervention” (“conditioned pain modulation” and “distraction by a cognitive task”), indicating significant interaction between “time” and “intervention”

During CPM, both PCES-induced pain and PCES-EP amplitude (Fig. 2a, b) significantly decreased during CS application (p < 0.001 for the within factor “time” for both PCES-induced pain intensity and PCES-evoked potentials, Tables 2 and 3).

**Table 2:** Repeated measures ANOVA for PCES-induced pain intensity with within factors “time” (“at baseline” and “during intervention”) and “intervention” (“conditioned pain modulation” and “distraction by a cognitive task”)

Within-subject factor	*F*-value	Significance	Partial *η*^2^
Time	F_1;22_ = 127.844	*p* < *0.001*	0.853
Intervention	F_1;22_ = 0.447	p = 0.511	0.020
Time * intervention	F_1;22_ = 0.662	p = 0.424	0.029

Italic values indicate significance of p-value (*p* < 0.01)

**Table 3:** Repeated measures ANOVA for amplitudes of PCES-evoked potentials within factors “time” (“at baseline” and “during intervention”) and “intervention” (“conditioned pain modulation” and “distraction by a cognitive task”)

Within-subject factor	F-value	Significance	Partial η^2^
Time	F_1;22_ = 50.362	*p* < *0.001*	0.696
Intervention	F_1;22_ = 1.077	p = 0.311	0.047
Time * intervention	F_1;22_ = 13.168	*p* = *0.001*	0.382

Italic values indicate significance of p-value (*p* < 0.01)

Only one subject showed no CPM-effect_PAIN_ (∆ NRS = 0), based on the pain ratings. Four subjects showed no CPM-effect_AMPLITUDE_, presenting with slightly increased amplitudes of PCES-EP during CS application.

**Fig. 3:**
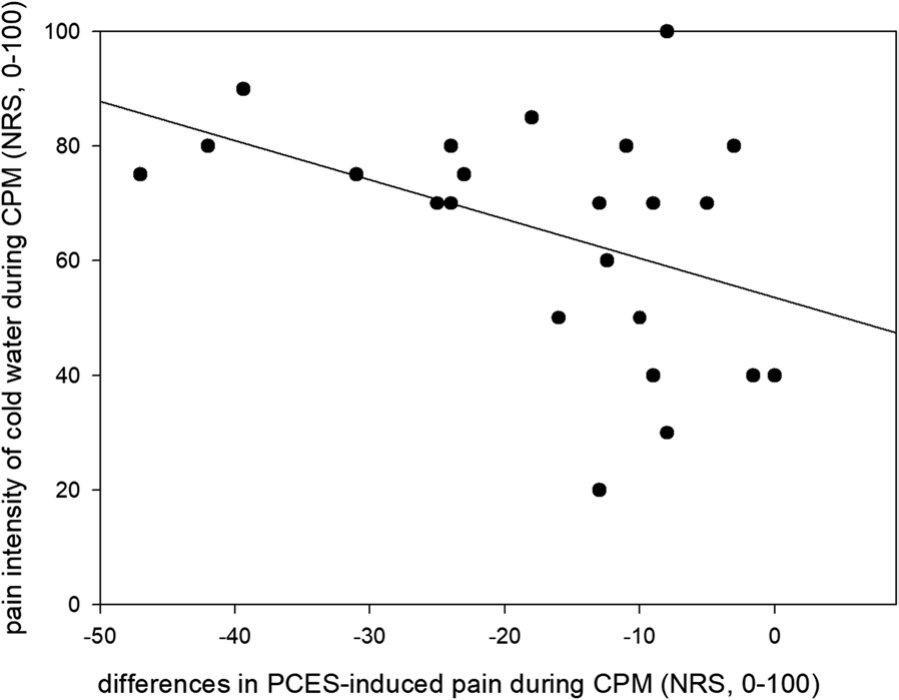
.

The pain intensity of cold water during conditioned pain modulation was rated with 65.2 ± 20.4 (range: 20–100) on the NRS (0–100). Pain ratings of the cold water correlated inversely with differences of PCES-induced pain (r = −  0.44, p = 0.037, Fig. 3), though a correction for multiple comparisons did not yield a significant result. There was no correlation between the pain ratings of the cold water and the ratios for PCES-EP amplitudes of the CPM intervention at all (r = − 0.069, p = 0.766).

The 15 subjects excluded from the further analysis due to artifacts during the PCES-recordings rated the pain intensity induced by the cold water on average significantly more severe compared to the 23 subjects included in the further analysis (NRS 78.0 ± 10.1, range 65–100, p = 0.031, unpaired t-test).

#### Effects of distraction

During distraction, in all subjects both PCES-EP amplitude and PCES-induced pain (Fig. 2a, b) decreased significantly during the 1-back task (p < 0.001 for the within factor “time” for both PCES-induced pain intensity and PCES-evoked potentials, Tables 2 and 3).

The perceived severity grade of the task was on average 4.9 ± 2.0 and the anger felt about doing a mistake during the task was 6.3 ± 2.2 (reported on the 11-point numerical rating scale 0–10). During distraction both severity grade and anger did not correlate with changes of PCES-EP amplitudes (r = 0.202, p = 0.312 and r = 0.044, p = 0.826, respectively) or PCES-induced pain (r = − 0.153, p = 0.446 and r = 0.111, p = 0.583, respectively).

### Comparison of the effects of both interventions

#### PCES-induced pain

ANOVA yielded a significant effect of within-subjects factor “time” on PCES-induced pain (p < 0.001), while there was no significant effect of the within-subject factor “intervention” on PCES-induced pain (see Table 2), indicating that the PCES-induced pain reduced significantly during the intervention but the reduction of pain intensity did not differ between CPM and distraction (Tables 1 and 2).

#### Amplitudes of PCES-EP

Analyzing amplitudes of PCES-EP, the ANOVA found a significant effect of the within-subject factor “time” (p < 0.001). Moreover, we found a significant interaction between the within-subject factors “intervention” and “time” regarding the amplitudes of PCES-EPs (p = 0.001). This indicates that the amplitudes changed significantly during both interventions, however the changes during distraction were more pronounced than those during CPM (Tables 1 and 3).

#### Correlations between changes during both interventions

There was no significant correlation between the changes during CPM and distraction by a cognitive task neither regarding the PCES-induced pain (r = − 0.18, p = 0.424), nor regarding the PCES-EP amplitudes (r = 0.11, p = 0.623).

### Relation between PCES-induced pain and PCES-EP amplitudes

Changes of the PCES-induced pain (difference) and the PCES-amplitudes (ratio) correlated significantly neither during the CPM session (r = − 0.32, p = 0.132) nor during the distraction session (r = 0.03, p = 0.885).

## Discussion

In summary, using the recently introduced novel CPM-paradigm we demonstrated that both PCES-induced pain and PCES-EP amplitudes can be reduced not only by CPM [20, 39] but also during distraction by a cognitive task. While the amount of pain relief induced by CPM and distraction by a cognitive task did not differ significantly during both interventions, the decrease of PCES-EP amplitudes after distraction by a cognitive task was slightly more pronounced than during CPM. Further on, the changes during both interventions did not correlate. The latter implies that both conditions might not represent the general pain modulatory capacity of individuals, but may underlie different neuronal mechanisms, having the perceived pain reduction as final common pathway.

Focusing on the effect on pain intensity, our findings are in line with recent studies, showing that both conditioned pain modulation [14, 20, 38, 39] and distraction [29–33] can reduce the perceived pain intensity. Various CPM paradigms have been published using different combination of stimuli as TS and CS, for review see [47]. With PCES as TS and cold water as CS we demonstrated a reduction of pain intensity during application of CS, which was accompanied by a significant decrease of PCES-EP amplitudes similar to our previous study [39]. The pain intensity of the cold water correlated with changes of PCES-induced pain; i. e. the more painful the conditioning stimulus was, the more pronounced it reduced the intensity of PCES-induced pain, though in contrast to the findings of our pilot study [39] the results were not significant after correction for multiple comparisons. Also, the significant correlation between pain intensity of the cold water and ratios of PCES-EP amplitudes [39] was not found in the present study. Further studies are needed to examine the influence of CS-induced pain on PCES-EPs.

Using the 1-back task, the magnitude of pain decrease in our study (on average 37%) was well within the range of previous studies using distraction models for pain modulation such as continuous cognitive visual task, memorization task, calculations task, high tech and low tech virtual reality, where the reduction of the pain ratings ranged from 13 to 50% [38, 48, 49].

Interestingly, the perceived difficulty of the 1-back task did not correlate with the effect of distraction on PCES-EPs and PCES-induced pain. This finding is in contrast to the above reported correlation between the intensity of CS and changes of PCES-induced pain during CPM; however, in accordance with a previous study, reporting no linear relation between the difficulty of the distraction task and the pain reduction [38]. In this study a moderate task was found to be more effective in reducing pain than a simple or difficult one. Another study reported even higher pain ratings and more pronounced spinal nociceptive responses measured by the nociceptive flexion reflex after the performance of tasks requiring high cognitive control compared to that with lower cognitive demand [50].⁠ Nevertheless, the fact that PCES-EP amplitudes were strongly affected by distraction suggests that higher cognitive circuits are involved in the generation of the PCES-evoked cortical potentials.

Sequential CPM paradigms are a clearer representation of descending inhibition since they are less biased by distraction of the painful CS. One might argue that our CPM protocol evaluating the TS changes during a parallel CS application might not be specifically enough for assessment of the descending pain inhibition. This might be true for the changes in pain intensity, which were similar during both interventions. However, some differences must be assumed, because while the changes in pain intensity correlated only with the perceived pain intensity of the CS, though this significant result was not yielded after correction for multiple comparisons, but there was no correlation at all with the perceived difficulty of the 1-back task. Also, the missing correlation of the changes during both interventions for both pain intensity and EP-amplitudes imply that the intraindividual capacity to modulate pain differ between both conditions. In addition, adding a distraction task simultaneously to the CS and proofing additional reduction of pain ratings and PCES-EPs amplitudes would have been a further argument for different acting mechanisms of the two interventions. Unfortunately, this was technically not possible during the current experiments. A previous study examining CPM based on brief heat stimuli as TS and tonic heat as CS and in comparison to continuous visual cognitive distracter tasks alone and in combination demonstrated an additive effect of CPM and distraction on pain inhibition, suggesting that CPM acts independently from distraction [38].

One important limitation of our study is that fifteen subjects had to be excluded from the analysis. Ten of them were excluded due to movement artifacts that were especially observed during the CPM assessment. Interestingly, the subjects excluded due to artifacts during recordings of PCES-EP rated the pain intensity of the CS higher than those with well identifiable PCES-EP, which might have biased our results. One possible explanation could be that muscle contractions in reaction to the painfully cold water led to movement artifacts and that the ten excluded subjects presented too many artifacts because they perceived the pain induced by the CS was too strong. Thus, this subjects’ group might represent subjects with generally higher pain sensitivity to cold stimuli. In conclusion, we suggest that cold water as CS should be painful enough to lead to an efficient pain inhibition/efficient CPM effect, but it should be considered for future studies using electrophysiological readouts that the more painful the CS was, the more movement artifacts due to muscular tensions occurred. Also after exclusion of subjects with artifacts in the PCES-EP recording, the studied cohort might have not been large enough to detect significant differences between the effect of conditioned pain modulation and distraction by a cognitive task. In terms of the correlations between the PCES-induced pain intensity, the amplitudes of the PCES-EPs and the intensity of the CS, further cumulative analysis of higher number of subjects are needed to further elucidate the question of a possible correlation between the differences in pain ratings (during intervention minus baseline) and the ratio of evoked potential amplitudes during conditioned pain modulation and the influence of any confounding factors.

Another possible limitation of our study was that the stimulus intensity needed to induce pain with intensity of 60 on the NRS (0–100) during the second intervention was higher than during the first one. This might be well explained by a habituation effect after PCES, as we previously demonstrated that repetitive PCES leads to a moderate reduction of PCES-induced pain, thus requiring slightly stronger stimulus intensities to achieve the required pain intensity of 60 on the NRS (0–100). Nevertheless, the CPM-effect was significantly larger than the effect of habituation on pain intensity and could therefore not be explained by habituation alone [20]. Furthermore, the significantly different stimulation intensity did not influence our results as we found no significant sequence effects on the outcome in the present study.

Further on, in the present study we focused on the direct comparison of painful cold water and the n-back-task and did not include control conditions for both CS, i.e. non-painful CS for CPM (e.g. immersion in 25 °C water) and passive viewing of stream of letters for cognitive task. We have recently reported significant differences between non-painful and painful CS regarding both CPM effects based on pain intensity and PCES-EP amplitudes [39], while EEG recording in combination for a control condition of the distraction task are missing. Therefore, it cannot be excluded that the somatosensory stimulation associated with immersing the hand in water (even without pain) and the visual stimulation associated with viewing letters (even without cognitive load) already modulate PCES-EP compared to a baseline PCES-EP at rest and that this modulation differs between the two conditions (somatosensory vs. visual stimulation). Thus, we cannot definitely rule out that the difference in PCES-EP amplitudes reduction between CPM and cognitive distraction can be at least partially attributed to this fundamental difference between the two interventions (somatosensory vs visual stimulation).

Some authors argue that the use of the concentric electrodes to record PCES-EPs as in the present study activates not selectively the nociceptive fibers [40, 41], but are mostly related to a large myelinated fiber input. In contrast, others have demonstrated that the peak-to-peak N1-P1-amplitudes assessed in a similar manner as in our study were reduced both in healthy controls and patients with neuropathic pain after application of capsaicin 8%, thus being a reliable A-delta test [51]. Anyway, the question regarding the selective activation of nociceptive fibers by using concentric electrodes for electrical stimulation was not in the scope in the present study. Moreover, considering previously reported CPM paradigms using pain induced by pressure or ischemic block as TS are also based on large fiber activation, therefore this should not be a relevant limitation for the comparison between the CPM-effect and effect of distraction on pain intensity.

## Conclusion

In conclusion, our findings displayed that both CPM and distraction are effective endogenous mechanisms for reduction of PCES-induced pain but also PCES-EP amplitudes. However, the even more pronounced decrease of PCES-EP amplitudes after distraction by a cognitive task and the missing correlation of the changes between both interventions imply that both conditions do not reflect the general pain modulatory capacity of individuals, but may underlie different neuronal mechanisms with the final common pathway of numerically perceived pain reduction.

Future studies recording PCES-EP not only above Cz but multi-segmentally at different levels of the afferent pathway (peripheral, spinal, cortical) and foot immersion as CS for heterosegmental activation or including also functional imaging might further elucidate the differences of their neural circuits.

## Data Availability

The datasets used and/or analyzed during the current study are available from the corresponding author on reasonable request.

## Abbreviations

CPM: Conditioned pain modulation
TS: Test stimulus
CS: Contact stimulus
PCES: Painful cutaneous electrical stimulation
EP: Evoked potentials
PCES-EP: Evoked potentials after painful cutaneous electrical stimulation
fMRI: Functional magnetic resonance imaging
DT: Detection threshold
PT: Pain thresholds
NRS: Numerical rating scale
